# Quality Evaluation of *Artemisia capillaris* Thunb. Based on Qualitative Analysis of the HPLC Fingerprint and UFLC-Q-TOF-MS/MS Combined with Quantitative Analysis of Multicomponents

**DOI:** 10.1155/2021/5546446

**Published:** 2021-04-21

**Authors:** Ying Dai, Zhihua Dou, Rongrong Zhou, Lin Luo, Li Bian, Yufeng Chen, Jinhua Tao, Zhixian Chen

**Affiliations:** ^1^School of Pharmacy, Nanjing University of Chinese Medicine, Nanjing 210023, Jiangsu, China; ^2^Nantong Third People's Hospital, Nantong University, Nantong 226006, Jiangsu, China; ^3^School of Pharmacy, Nantong University, Nantong 226019, Jiangsu, China; ^4^Department of Pharmacy, Nantong Health College of Jiangsu Province, Nantong 226010, Jiangsu, China

## Abstract

In this study, a new method was developed for the comprehensive quality evaluation (QE) of *Artemisia capillaris* Thunb. (*A. capillaris*, named Yinchenhao in Chinese), which is one of the most commonly used herbal medicines (HMs). First, fingerprints of 31 batch samples of *A. capillaris* were determined by HPLC, the reference fingerprint was established, and the common peaks were assigned. Second, the components of common peaks in the HPLC fingerprints were identified by ultrafast liquid chromatography- (UFLC-) Q-TOF-MS/MS. Finally, the contents of the components unambiguously confirmed by reference substances were determined, and the correlation between the contents of chlorogenic acid and the contents of others was analyzed. The results showed that there were 20 common peaks in the HPLC fingerprints of 31 batch samples. The components of these 20 common peaks were identified as ten organic acids, eight flavonoids, and two others. Among nine organic acids such as 1-caffeoylquinic acid, neochlorogenic acid, chlorogenic acid, caffeic acid, cryptochlorogenic acid, 1,3-dicaffeoylquinic acid, 3,4-dicaffeoylquinic acid, 3,5-dicaffeoylquinic acid, and 4,5-dicaffeoylquinic acid, three flavonoids such as rutin, hyperoside, and isoquercetin, and one other *p*-hydroxyacetophenone, a total of 13 ones were unambiguously identified by comparison with reference substances; one caffeoylquinic acid glucoside and one flavone di-C-glucoside were detected in *A. capillaris* for the first time. There were some differences in the contents of 13 components in different samples; chlorogenic acid could be regarded as the quality marker of *A. capillaris*. The current established method in this study can be used for the comprehensive QE of *A. capillaris* and can also provide reference for the QE of the other HMs.

## 1. Introduction

The quality and quality evaluation (QE) method is crucial in the effectiveness and safety assessment of herbal medicines (HMs) [1]. The method for the QE of TCMs must be based on the holistic principle, and fingerprint describes integral characterization and reflects interactive aspects of complex components; therefore, it can offer the possibility of evaluating quality of HMs following the overall principle [2]. HPLC fingerprint has become the most widely used method due to its high reproducibility and sensitivity [3]. The HPLC fingerprint method applied to QE of HMs is mainly based on the similarity of fingerprints and the presence or absence of chromatographic peaks among samples [4, 5]; however, this method cannot identify what these peaks are. Q-TOF-MS/MS is a kind of tandem mass spectrometry providing a high mass resolution and accurate mass measurement for the structural elucidation of unknown chemicals [6] and can be used in the identification of common peaks in HPLC fingerprints. Based on the qualitative analysis of fingerprint, the quantitative analysis of multiple components is the key step of QE of HMs [5].

*Artemisia capillaris* Thunb. (*A. capillaris*, named Yinchenhao in Chinese) is one of the most commonly used HMs [7], which has been used in China, Korea, and Japan for a long time to treat liver and choleretic disorders, such as cholestasis, jaundice, liver fibrosis, and hepatitis [7–9]. The major components contained in *A. capillaris* include organic acids, flavonoids, coumarins, essential oil, and others, such as *p*-hydroxyacetophenone [7]. A characteristic fingerprint was developed to determine the volatile constituents in essential oil of *A. capillaris* by GC-MS [10], but the systematic study on the HPLC fingerprint of *A. capillaris* and the identification of common peaks by Q-TOF-MS have not been reported so far. In recent years, QE of *A. capillaris* based on multicomponents quantitative analysis has made some progress. Yu et al. developed a method to determine eight organic acids in *A. capillaris* extract by HPLC, including chlorogenic acid (CA), neochlorogenic acid (NCA), cryptochlorogenic acid (CCA), 1,3-dicaffeoylquinic acid (1,3-diCQA), 3,4-dicaffeoylquinic acid (3,4-diCQA), 3,5-dicaffeoylquinic acid (3,5-diCQA), 4,5-dicaffeoylquinic acid (4,5-diCQA), and caffeic acid [11]. Tian et al. established a quantitative analysis method of six organic acids of NCA, CA, CCA, 1,3-diCQA, 3,4-diCQA, and 4,5-diCQA in *A. capillaris* and its decoction by HPLC [12]. Thirteen components including four organic acids, four flavonoids, four coumarins, and one other of *p*-hydroxyacetophenone and ten components including four organic acids, five flavonoids, and one coumarin of scoparone in *A. capillaris* were determined by the same method, respectively [13, 14]. However, a comprehensive QE method for *A. capillaris* has not been established so far. Therefore, the aim of this work was to establish a new method for the QE of *A. capillaris* comprehensively based on qualitative analysis of the HPLC fingerprint and ultrafast liquid chromatography- (UFLC-) Q-TOF-MS/MS combined with quantitative analysis of multicomponents.

## 2. Experimental

### 2.1. Chemicals and Reagents

Reference substance of *p*-hydroxyacetophenone (no. 111897–201602, with purity ≥99.9%) was purchased from the National Institutes for Food and Drug Control (Beijing, China). 1-caffeoylquinic acid (1-CQA, no. CHB170525), NCA (no. CHB170914), CA (no. CHB170713), caffeic acid (no. CHB160907), CCA (no. CHB170828), 1,3-diCQA (no. CHB160620), rutin (no. CHB170303), hyperoside (no. CHB160904), isoquercetin (no. CHB160912), 3,4-diCQA (no. CHB160725), 3,5-diCQA (no. CHB171013), and 4,5-diCQA (No. CHB160726) were purchased from the Chengdu Chroma Biotechnology Co., Ltd. (Chengdu, China) (all with purity ≥98%). Methanol (HPLC grade) and acetonitrile (LC/MS grade) were purchased from Fisher Scientific (Fair Lawn, NJ, USA). Purified water was purchased from Wahaha Group Co., Ltd. (Hangzhou, China). Formic acid (HPLC grade) was supplied by Nanjing Chemical Reagent Co. Ltd. (Nanjing, China).

### 2.2. Apparatus

Determination of the HPLC fingerprint and the contents of 13 components were performed on a HPLC system (Waters Corp., Milford, MA, USA), equipped with a Waters e2695 separation unit, a Waters 2998 PDA detector, and an Empower 3 data processing system. Chromatographic separation was performed on a Symmetry C_18_ column (4.6 mm × 250 mm, 5 *μ*m, Waters Corp., USA). Acetonitrile (*A*) and 0.1% (*v*/*v*) formic acid (B) were used as mobile phases with the following gradient elution: 0−35 min, 5−10% A; 35−65 min, 10−25% A; 65−67 min, 25−90% A; and 67−80 min, 90% A. The flow rate was set at 1.0 mL/min, and the column temperature was maintained at 30°C. The injection volume of sample solution was 30 *µ*L. The detection wavelength of fingerprint and content of *p*-hydroxyacetophenone, rutin, hyperoside, and isoquercetin was set at 254 nm and that of content of nine organic acids was set at 324 nm.

Identification of the common peaks in the HPLC fingerprint was performed on a UFLC-Q-TOF-MS/MS system. Separation was performed on a UFLC system (Shimadzu, Kyoto, Japan) by using a Symmetry C_18_ column (250 mm × 4.6 mm, 5 *μ*m); with the same mobile phases and the same gradient conditions abovementioned, the injection volume of the mixed reference substances solution for qualitative analysis and sample solution was all 20 *µ*L. After separation, mass spectra were acquired on the AB Triple TOF 4600 plus system (AB SCIEX, Framingham, USA) with the following mass spectrometric parameters: ion source, DuoSpray; ESI mode, negative; ion source temperature, 550°C; ion spray voltage, −4500 V; nebulizer gas (gas 1), 60 psi; heater gas (gas 2), 60 psi; and curtain gas (CUR), 35 psi. The TOFMS-IDA-10MS/MS information acquisition method was used to obtain mass spectrometry information, and the parameters were set as follows: decluster potential (DP) of −80 V, collision energy (CE) of −10 eV, accumulation time of 250 ms, mass range of 105–1500 Da for the TOF-MS scan, collision energy (CE) of −35 eV, collision energy spread (CES) of 15 eV, and mass range of 50–1500 Da for the TOF-MS/MS detection. LC-MS/MS data were analyzed using PeakView mass spectrometry analysis software (Version 1.6, AB SCIEX, USA).

### 2.3. Samples and Sample Preparation

Information on all 31 batches of samples is given in Table 1, among which, 30 batches of *A. capillaris* (S1–S30) were purchased from different large TCM hospitals in China and authenticated as the dried aerial part of *A. capillaris* by the chief Chinese pharmacist Xudong Gong, the director of the Nantong Food and Drug Supervision and Inspection Centre. Herbal reference substance of *A. capillaris* (S31) was purchased from the National Institutes for Food and Drug Control (Beijing, China) in 2018.

The samples were dried at 40°C, ground into powder, and then sieved through a 40-mesh screen. Approximately 0.2 g of sample powder was accurately weighed and placed in a 50 mL dark brown volumetric flask. Approximately 49 mL of 50% (*v*/*v*) methanol was added and extracted by ultrasonication (200 W, 53 kHz) for 30 min. After cooling to room temperature, 50% (*v*/*v*) methanol was added for calibration of the volumetric flask and shaken well. The extract was filtered through a 0.22 *μ*m filter membrane, and the filtrate was taken as the sample solution.

### 2.4. Preparation of Reference Substance Solutions

Appropriate amounts (5–20 mg) of 13 reference substances were accurately weighted, dissolved with 50% (*v*/*v*) methanol, respectively, and 13 reference substance stock solutions were prepared.

The mixed reference substances solution for qualitative analysis with a concentration range of 0.4–50 *μ*g/mL of each compound was prepared by accurately absorbing appropriate volume of 13 reference substance stock solutions, mixing them, and diluting the mixture with 50% (*v*/*v*) methanol.

Working solution A for quantitative analysis was prepared by the same method as the mixed reference substances solution for qualitative analysis, and the final concentrations of 13 reference substances were at the range of 3.1–193 *μ*g/mL. Working solution A was diluted two, five, and ten times with 50% (*v*/*v*) methanol to prepare working solutions B, C, and D, respectively.

### 2.5. Method Validation of the HPLC Fingerprint Analysis

The method of HPLC fingerprint determination was validated with precision, stability, and repeatability tests, by using peak 5 (CA) as the reference peak and the relative standard deviation (RSD) value of the average relative retention time (RRT) and relative peak area (RPA) of the 20 common peaks as measure values. In the precision test, six consecutive injections of the same sample (S1) solution were analyzed. Stability was examined by analyzing the sample solution (S1) at 0, 6, 12, 18, 24, and 36 h after preparation. Repeatability was examined by determination of six sample solutions prepared in parallel from S1.

### 2.6. Method Validation of the Quantitative Analysis

The method of quantitative analysis was validated with investigation of linear relationships, limit of quantitation (LOQ), limit of detection (LOD), precision, stability, repeatability, and the recovery test of 13 components. Investigation of linear relationships was performed by precisely injecting working solutions B, C, and D 10 *μ*L and working solution A 10, 20, 30, and 40 *μ*L into the HPLC systems for the calculation of the regression equations, correlation coefficients, and linear ranges of 13 components. Working solution D was successively diluted with 50% (*v*/*v*) methanol to give different concentrations of reference substance solutions. The LOQ and LOD values were determined by using signal-to-noise ratios of 3 : 1 and 10 : 1 and injecting 10 *μ*L of above different concentrations of reference substance solutions. By using the RSDs of the peak areas of the 13 components as the measurement values, intraday precision, interday precision, and stability tests were performed, respectively. In the intraday precision test, six consecutive injections of 30 *μ*L working solution A were analyzed, and in the interday precision test, six injections of 30 *μ*L working solution A were analyzed twice a day over three consecutive days. Stability was examined by analyzing the peak areas of nine organic acids at 324 nm and four others at 254 nm detected in Section 2.5 of the stability test. Repeatability was examined by calculating the contents of 13 components according to the peak areas of nine organic acids at 324 nm and four others at 254 nm detected in Section 2.5 of the repeatability test and using the RSDs of the contents as the measured values. In the recovery test, approximately 0.1 g of S1 powder was weighed precisely, and then, 13 reference substance stock solutions were added to the sample in a certain volume according to the approximate proportion of the sample content to the reference substance (1 : 1) to prepare six sample solutions in parallel. The six sample solutions were injected into HPLC, and the average recovery rates and RSDs of the 13 components were calculated.

We followed the methods of Author links open overlay panel [15].

## 3. Results and Discussion

### 3.1. Method Validation of the HPLC Fingerprint Analysis

The RSDs of RRT and RPA for precision were less than 0.10% and 4.4%, those of stability were no more than 0.08% and 4.7%, and those of repeatability did not exceed 0.08% and 4.8%, respectively. The results met the national standards of TCM fingerprinting [16].

### 3.2. Method Validation of the Quantitative Analysis

As given in Table 2, the high correlation coefficient values (*R*^2^ ＞ 0.9998) displayed good linearity over a wide range of injected amounts, and as given in Table 3, the RSDs of the intraday precision, interday precision, stability, and repeatability were all less than 5%, and the average recovery rates were in the range of 95.49%–103.20% with RSD values ranging 0.90–4.95%. The above results met the requirements of drug quality standard analysis method in Chinese Pharmacopoeia [17].

### 3.3. Establishment of the HPLC Fingerprint and Similarity Analysis

**Figure 1:**
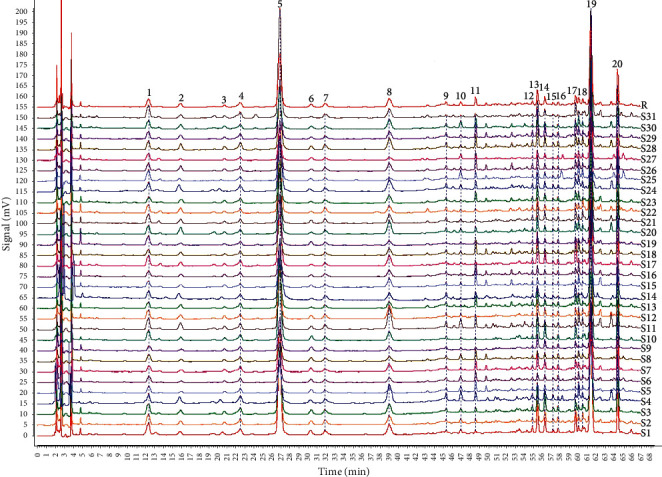
Chromatograms of 31 batches of *A. capillaris* (S1–S31) and the reference chromatogram (R).

31 batches of *A. capillaris* samples were determined (chromatograms are shown in Figure 1). The chromatographic data of the samples were imported into the software of Similarity Evaluation System for Chromatographic Fingerprint of Traditional Chinese Medicine (version 2012, Chinese Pharmacopoeia Commission, Beijing, China). Using the chromatogram of S1 as a reference, the reference chromatogram was generated, and 20 peaks were extracted to be the common peaks (shown in Figure 1). The similarities between sample chromatograms and reference chromatogram were calculated by the above software, and the results showed that the similarities of 31 batches of *A. capillaris* were all greater than 0.9 (Table 3).

### 3.4. Identification of the Common Peaks by UFLC-Q-TOF-MS/MS

**Figure 2:**
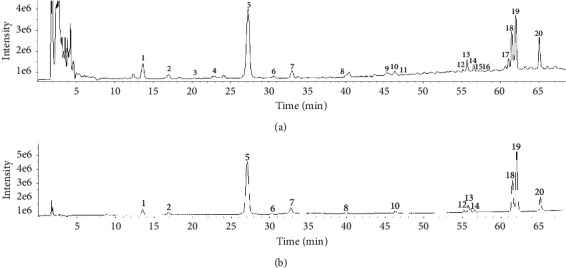
Total ion chromatogram of *A. capillaris* sample (a) and mixed reference substances (b) (negative ion mode).

Since more information and higher identifiability of fragmentation was observed in the negative ion mode, it was chosen for MS analysis rather than the positive ion mode. First, total ion chromatograms of the *A. capillaris* sample and mixed reference substances (Figure 2) were extracted using PeakView mass spectrometry analysis software. Second, the mass spectral data and dissociative rules of the reference substances were summarized, and the law of the quasimolecular ion [M − H]^−^ and/or [M + Cl]^−^ that could be selected as the precursor ion for collision-induced dissociation fragmentation to produce MS/MS product ions spectra was revealed. Finally, the components of the total 20 common peaks in the HPLC fingerprint were identified by comparing the retention time, *m*/*z* of [M − H]^−^ and/or [M + Cl]^−^ and MS/MS fragmentation patterns with those of the reference substances or previous literature reports, combining with online retrieval of two compound database of PubChem (http://pubchem.ncbi.nlm.nih.gov) and ChemSpider (http://www.chemsipider. com). The mass spectral data are given in Table 4.

Among 20 components, 13 ones were unambiguously identified by comparison with the reference substances, including nine organic acids such as 1-CQA (peak 1), NCA (peak 2), CA (peak 5), caffeic acid (peak 6), CCA (peak 7), 1,3-diCQA (peak 10), 3,4-diCQA (peak 18), 3,5-diCQA (peak 19), and 4,5-diCQA (peak 20), three flavonoids such as rutin (peak 12), hyperoside (peak 13), and isoquercetin (peak 14), and one other *p*-hydroxyacetophenone (peak 8).

For peak 3, the molecular formula of C_19_H_26_O_11_ was speculated by software, and its quasimolecular ion was at an *m*/*z* of 465.1162 ([M* *+* *Cl]^−^). No literature reported the compounds with the molecular formula of C_19_H_26_O_11_ in *A. capillaris* so far. Sixty-nine and twenty-seven compounds consistent with this formula were retrieved from PubChem and ChemSpider, respectively. The structures of these compounds were analyzed one by one by the exclusion method, combined with *p*-hydroxyacetophenone and 6′-*O*-dicaffeoyl-*p*- hydroxyacetophenone-4-*O*-*β*-D-glucoside, and another compound with same parent nucleus [18], existed in *A. capillaris*; peak 3 was temporarily identified as 4-acetylphenyl 6-*O*-*β*-D-xylosyl-*β*-D-glucoside (6′-*O*-xylosyl-*p*-hydroxyacetophenone-4-*O*-*β*-D-glucoside), the first compound in both databases. In MS/MS spectrum of this compound, *m*/*z* of 465.1170, 429.1407, 329.0680, 293.0897, and 135.0455 were determined, which was corresponded to [M* *+* *Cl]^−^, [M-H]^−^, [M* *+* *Cl]^−^ loss of *p*-hydroxyacetophenone (C_8_H_8_O_2_), [M* *−* *H]^−^ loss of C_8_H_8_O_2_, and [M* *−* *H]^−^ loss of xylose-glucosyl (C_11_H_18_O_9_), respectively.

According to literature [19], the component of peak 4 was identified as a caffeoylquinic acid glucoside. The quasimolecular ion of this component was at an *m*/*z* of 515.1409 ([M* *−* *H]^−^), and the MS/MS fragment ions were determined as an m/*z* of 515.1426 corresponding to [M-H]^−^, an *m*/*z* of 353.0868 corresponding to [515-C_6_H_10_O_5_ (glucosyl)]^−^, an *m*/*z* of 323.0768 corresponding to [515-C_7_H_12_O_6_ (quinic acid)]^−^, an m/*z* of 191.0561 corresponding to [353-C_9_H_6_O_3_ (caffeyl)]^−^, an *m*/*z* of 179.0343 corresponding to [353-C_7_H_10_O_5_ (residue of quinic acid)]^−^, and an *m*/*z* of 161.0241 [353-C_7_H_12_O_6_]^−^. The linkage position between caffeoyl and quinic acid could be distinguished based on the MS^2^ fragmentation; when this position was at 1-OH, 3-OH, or 5-OH, the *m*/*z* of 191 was the base peak; while linkage position was at 4-OH, the *m*/*z* of 173 was the base peak [19]. An *m*/*z* of 191 was determined as the base peak of peak 4, so the connection position of 4-OH was excluded. The relative intensity of *m*/*z* 179 fragment ion could also be used to determine the linkage position between caffeoyl and quinic acid [19]. The relative intensity of *m*/*z* 179 fragment ion of peak 4 was determined as 5.62%, the one of 1-CQA (peak 1) and NCA (peak 2, linkage position was at 5-OH) was determined as 6.06% and 55.28%, respectively, and fragment ion of *m*/*z* 179 was not detected in CA (peak 5, linkage position was at 3-OH). Therefore, the component of peak 4 was temporarily identified as 1-*O*-(4′-*O*-*β*-D-glucosyl caffeoyl) quinic acid or 1-*O*-(3′-*O*-*β*-D-glucosyl caffeoyl) quinic acid, which was first detected in *A. capillaris*, to the best of our knowledge.

**Figure 3:**
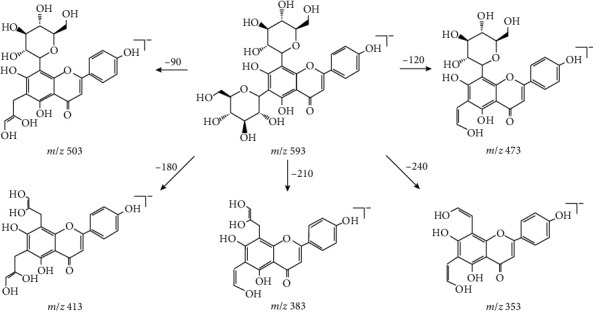
The possible dissociation pathway of the component of peak 9.

The component of peak 9 was a typical flavone di-*C*-glucoside, according to the analysis of detected mass spectrometry data and literature [20]. The quasimolecular ion of this component was determined as an *m*/*z* of 593.1535 ([M-H]^−^), and the MS/MS fragment ions were *m*/*z* of 593.1553, 503.1209, 473.1101, 413.0927, 383.0784, and 353.0674, which were consistent with the mass spectrum data of apigenin 6,8-di-*C*-*β*-D-glucoside reported in the literature [20], and its possible dissociation pathway is shown in Figure 3. To the best of our knowledge, this component was also first detected in *A. capillaris*.

The component of peak 11 was identified as the isomer of rutin according to literature [21]. Its quasimolecular ion was determined as an *m*/*z* of 609.1456 ([M-H]^−^) and an *m*/*z* of 645.1207 ([M + Cl]^−^), and its MS/MS fragment ions were *m*/*z* of 609.1480, 447.0915, and 301.0339, which correspond to [M-H]^−^, [609-glucosyl]^−^, and [609-C_12_H_20_O_9_ (rutinose)]^−^, respectively.

The components of peak 15, 16, and 17 were temporarily identified as three flavonoids of kaempferol-3-*O*-glucoside, kaempferol-3-*O*-rutinoside (nicotiflorin), and quercetin-3-*O*-rhamnoside [22, 23], respectively. The quasimolecular ions of peak 15 were at *m*/*z* of 447.0945 ([M-H]^−^), the ones of peak 16 were determined as *m*/*z* of 593.1510 ([M-H]^−^), and the ones of peak 17 were determined as *m*/*z* as 447.0939 ([M-H]^−^) and 483.0705 ([M + Cl]^−^). The MS/MS fragment ions of peak 15 were *m*/*z* of 447.0940 due to [M-H]^−^ and 285.0404 due to [M-H-glucosyl]^−^, the ones of peak 16 were *m*/*z* of 593.1525 and 285.0469, and the ones of peak 17 were *m*/*z* of 447.0954 and 285.0420.

**Figure 4:**
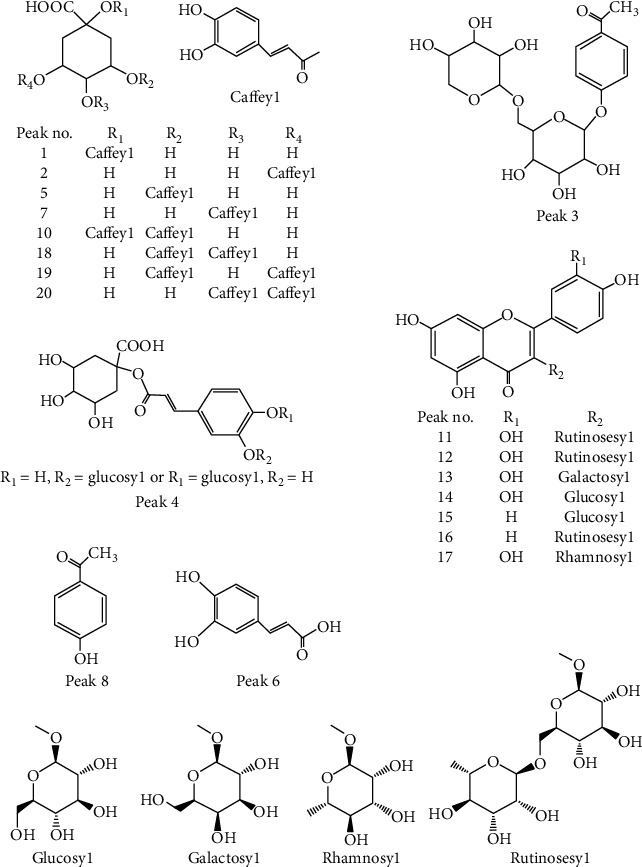
Structures or possible structures of the components of peaks 1–8 and peaks 10–20.

The structures or possible structures of the components of peaks 1–8 and peaks 10–20 are shown in Figure 4.

### 3.5. Wavelength Selection for Quantitative Analysis of 13 Components

**Figure 5:**
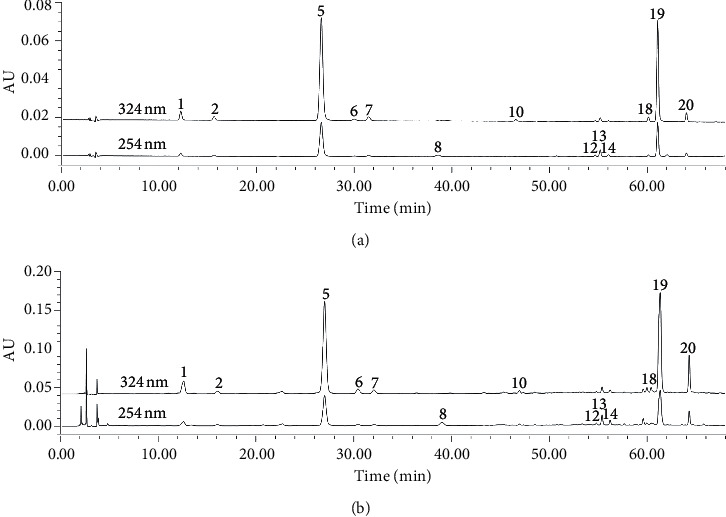
HPLC chromatograms of the mixed reference substances (a) and *A. capillaris* sample (b). The number of peaks is the same as Table 4.

It was found that all 13 components could be detected at 254 nm, but the peak of 3,4-diCQA (peak 18) has not been completely separated from the nearby ones; organic acids had strong absorption near 324 nm, but there was almost no absorption of *p*-hydroxyacetophenone (peak 8) at this wavelength. Therefore, 324 nm was selected as the detection wavelength for nine organic acids, and 254 nm was selected for other four components. The chromatograms of the mixed reference substances and sample are shown in Figure 5.

### 3.6. Contents of 13 Components in 31 Batches of *A. capillaris*

**Figure 6:**
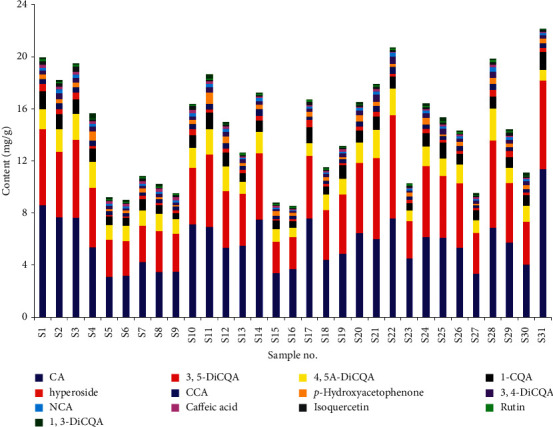
The bar graph of contents of 13 components in 31 batches of *A. capillaris*.

As given in Table 5 and Figure 6, there were some differences in the contents of 13 components in different samples, which is basically consistent with the previous literature reports [12–14]; however, the content of CA seems to have a certain correlation with the other 12 components and the total of 13 components. Therefore, the bivariate correlation analysis method in SPSS 20 statistical software was used to analyze the correlation between the contents of CA and the contents of 12 other components and the total content of all 13 components in 31 batches of *A. capillaris*. As the results given in Table 6, the contents of 10 components and the total content of 13 components were significantly correlated with the content of CA (*P* < 0.01 or *P* < 0.05), except for the content of caffeic acid and 1,3-diCQA, which had poor correlation with the content of CA (*P* > 0.05). According to the data in Table 5, the average contents of caffeic acid and 1,3-diCQA in 31 batches of samples only account for 1.10% and 0.92% of the average contents of all 13 components, respectively, so these two components can be ignored to a certain extent. According to the above analysis, the content of other components in different batches of *A. capillaris* is obviously related to the content of CA, that is to say, the content of CA largely reflects the quality of *A. capillaris*. Therefore, CA can be regard as the quality marker of *A. capillaris*. CA is the content determination component of *A. capillaris* in Chinese Pharmacopoeia. It is suggested that hospitals, pharmacies, and pharmaceutical manufacturers purchase multiple batches of *A. capillaris* and mix the high and low CA content batches before use according to the CA detection report provided by the supplier, so as to ensure clinical safety and effectiveness.

## 4. Conclusion

In this study, a new method was developed for the comprehensive QE of *A. capillaris* based on qualitative analysis of the HPLC fingerprint and UFLC-Q-TOF-MS/MS combined with quantitative analysis of multicomponents. The results showed that there were 20 common peaks in the HPLC fingerprints of *A. capillaris*. The similarities between the sample chromatograms and reference chromatogram were good. The components of the 20 common peaks were identified as ten organic acids, eight flavonoids, and two others. Among nine organic acids such as 1-CQA, NCA, CA, caffeic acid, CCA, 1,3-diCQA, 3,4-diCQA, 3,5-diCQA, and 4,5-diCQA, three flavonoids such as rutin, hyperoside, and isoquercetin, and one other *p*-hydroxyacetophenone, a total of 13 components were unambiguously identified by comparison with reference substances; one caffeoylquinic acid glucoside of 1-*O*-(4′-*O*-*β*-D-glucosyl caffeoyl) quinic acid or 1-*O*-(3′-*O*-*β*-D-glucosyl caffeoyl) quinic acid and one flavone di-*C*-glucoside of apigenin 6,8-di-*C*-*β*-D-glucoside were detected in *A. capillaris* for the first time. There were some differences in the contents of 13 components in different samples; chlorogenic acid could be regarded as the quality marker of *A. capillaris*. In summary, the method established in the present study can be used for the comprehensive QE of *A. capillaris* and can also provide reference for QE of other HMs.

## Figures and Tables

**Table 1:** Samples information and similarities.

Sample no.	Manufacturers	Batch no.	Origins of herb	Similarity
S1	Nantong Sanyue Herbal Pieces Co., Ltd.	171208	Henan	0.949
S2	Nantong Sanyue Herbal Pieces Co., Ltd.	180109	Henan	0.993
S3	Nantong Sanyue Herbal Pieces Co., Ltd.	151110	Henan	0.996
S4	Nantong Sanyue Herbal Pieces Co., Ltd.	180308	Henan	0.905
S5	Suzhou Tianling Herbal Pieces Co., Ltd.	180205	Jiangsu	0.958
S6	Suzhou Tianling Herbal Pieces Co., Ltd.	180205010	Jiangsu	0.963
S7	Suzhou Tianling Herbal Pieces Co., Ltd.	170902010	Jiangsu	0.980
S8	Suzhou Tianling Herbal Pieces Co., Ltd.	180205015	Jiangsu	0.969
S9	Suzhou Tianling Herbal Pieces Co., Ltd.	160309010	Jiangsu	0.963
S10	Suzhou Tianling Herbal Pieces Co., Ltd.	160122010	Jiangsu	0.990
S11	Yancheng Herbal Pieces Co., Ltd.	2017091402	Jiangsu	0.986
S12	Anhui Bozhou Qiancao Guoyao Co., Ltd.	1712176	Shanxi	0.991
S13	Anhui Wansheng Herbal Pieces Co., Ltd.	171201	Shanxi	0.994
S14	Anhui Wansheng Herbal Pieces Co., Ltd.	180302	Shanxi	0.928
S15	Anhui Mengshi Herbal Pieces Co., Ltd.	170401	Anhui	0.961
S16	Bozhou Qiaocheng Wanshixiang Herbal Pieces Co., Ltd.	170901	Shanxi	0.970
S17	Anhui Xiehecheng Pharmaceutical Herbal Pieces Co., Ltd.	17051814	Shanxi	0.986
S18	Anhui Shenghaitang Herbal Pieces Co., Ltd.	2017080421	Shanxi	0.991
S19	Hebei Kaida Pharmaceutical Co., Ltd.	20171201	Hebei	0.987
S20	Zhejiang Tongjuntang Herbal Pieces Co., Ltd.	170921	Shaanxi	0.996
S21	Zhengzhou Ruilong Pharmaceutical Co., Ltd.	17110102	Henan	0.990
S22	Hebei Renxin Pharmaceutical Co., Ltd.	21118001	Hebei	0.983
S23	Shaohuatang Guoyao Co., Ltd.	171010	Shanxi	0.975
S24	Shanghai Wanshicheng Guoyao Products Co., Ltd.	180418-1	Shandong	0.934
S25	Shanghai Kangqiao Herbal Pieces Co., Ltd.	171125	Shaanxi	0.972
S26	Yancheng Herbal Pieces Co., Ltd.	2016030202	Jiangsu	0.994
S27	Hangzhou Huadong Herbal Pieces Co., Ltd.	1601118	Shandong	0.953
S28	Bozhou Yonggang Herbal Pieces Co., Ltd.	15113001	Shanxi	0.991
S29	Bozhou Yonggang Herbal Pieces Co., Ltd.	15070901	Shanxi	0.994
S30	Yunnan Baiyao Herbal Medicine Branch of Yunnan Group Co., Ltd.	YP20140601	Hubei	0.983
S31	National Institutes for Food and Drug Control	121555-201101	Unknown	0.968

**Table 2:** Results of the investigation of the linear relationship, LOD, and LOQ.

Reference substance	Regression equation	*R* ^2^	Linear range (ng)	LOD (ng)	LOQ (ng)
1-CQA	*Y* = 2231204*X* − 8098	0.9999	13.35 ∼ 534	1.60	5.34
NCA	*Y* = 2830658*X* − 5170	0.9999	5.15 ∼ 206	0.34	1.72
CA	*Y* = 2870573*X* − 117405	0.9999	192.3 ∼ 7692	1.54	7.69
Caffeic acid	*Y* = 4958993*X* − 7629	0.9998	1.90 ∼ 76.05	0.30	1.52
CCA	*Y* = 2656572*X* − 12630	0.9998	7.66 ∼ 306.24	0.51	2.55
*p*-Hydroxyacetophenone	*Y* = 2885362*X* − 7875	0.9999	3.05 ∼ 121.92	0.41	1.63
1,3-DiCQA	*Y* = 2995412*X* − 1885	0.9999	1.77 ∼ 70.84	0.24	1.18
Rutin	*Y* = 1638734*X* − 1384	0.9999	2.53 ∼ 101	0.40	1.68
Hyperoside	*Y* = 2406676*X* − 5708	0.9999	8.31 ∼ 332.4	0.33	1.66
Isoquercetin	*Y* = 1973858*X* − 1855	0.9999	2.90 ∼ 116	0.23	1.74
3,4-DiCQA	*Y* = 2669900*X* − 7344	0.9999	5.04 ∼ 201.6	0.67	2.69
3,5-DiCQA	*Y* = 3267110*X* + 5860	0.9999	122.30 ∼ 4892	1.73	3.46
4,5-DiCQA	*Y* = 2863719*X* − 7095	0.9999	13.65 ∼ 546	1.09	5.46

**Table 3:** Results of precision, stability, repeatability, and recovery tests (*n* = 6).

Components	Precision RSD (%)		Stability RSD (%)	Repeatability (RSD, %)	Recovery	
	Intraday	Interday			Mean (%)	RSD (%)
1-CQA	0.51	0.37	0.71	2.16	101.23	2.11
NCA	0.66	0.69	2.26	3.82	97.68	0.90
CA	0.53	0.52	0.26	4.17	96.19	3.09
Caffeic acid	0.73	0.78	1.14	1.17	97.41	2.59
CCA	0.58	0.49	1.03	3.93	99.66	4.89
*p*-Hydroxyacetophenone	0.67	0.70	1.20	1.83	97.15	3.00
1,3-DiCQA	0.72	0.76	2.30	3.64	95.49	4.78
Rutin	2.84	0.92	3.59	2.22	96.68	4.32
Hyperoside	0.65	0.56	0.35	3.97	95.75	4.77
Isoquercetin	0.86	0.78	2.85	4.06	98.47	4.95
3,4-DiCQA	0.62	0.62	4.69	1.16	103.20	4.85
3,5-DiCQA	0.62	0.65	0.71	4.02	98.96	3.75
4,5-DiCQA	0.60	0.61	0.53	3.81	96.83	4.82

**Table 4:** Identification of the common peaks in the *A. capillaris* fingerprint by UHLC-Q-TOF-MS/MS.

Peak no.	*t*_R_ (min)	Formula	MS			MS/MS^c^	Identification	Types of compounds
			Measured	Theoretical	Error (ppm)			
1	13.525	C_16_H_18_O_9_	353.0885^a^	353.0878	2.0	191.0573	1-CQA^d^	Organic acids

2	16.933	C_16_H_18_O_9_	353.0885^a^	353.0878	2.0	191.0572, 179.0350, 135.0441, 353.0906	NCA^d^	Organic acids
			389.0631^b^	389.0645	-3.6	353.090		

3	20.198	C_19_H_26_O_11_	465.1162^b^	465.1169	-1.5	465.1170, 135.0455, 429.1407, 329.0680, 293.0897	6′-*O*-Xylosyl-*p*-hydroxyacetophenone-4-*O*-*β*-D-glucoside	Others

4	22.909	C_22_H_28_O_14_	515.1409^a^	515.1406	0.5	191.0561, 323.0768, 515.1426, 161.0241, 353.0868, 179.0343	1-*O*-(4′-*O*-*β*-D-Glucosyl caffeoyl) quinic acid or 1-*O*-(3′-*O*-*β*-D-glucosyl caffeoyl) quinic acid	Organic acids

5	27.281	C_16_H_18_O_9_	353.0886^a^	353.0878	2.2	191.0577	CA^d^	Organic acids

6	30.533	C_9_H_8_O_4_	179.0358^a^	179.0350	4.6	135.0456, 107.0505, 179.0348, 117.0340	Caffeic acid^d^	Organic acids

7	32.916	C_16_H_18_O_9_	353.0883^a^	353.0878	1.4	173.0462, 191.0568, 179.0355, 135.0461, 353.0901	CCA^d^	Organic acids
			389.0647^b^	389.0645	0.3	173.0457, 179.0354, 191.0568, 353.0885, 135.0456		

8	39.703	C_8_H_8_O_2_	135.0457^a^	135.0452	4.0	93.0354, 135.0457	*p*-Hydroxyacetophenone^a^	Others

9	45.515	C_27_H_30_O_15_	593.1535^a^	593.1512	3.9	593.1553, 353.0674, 473.1101, 383.0784, 503.1209, 413.0927	Apigenin 6, 8-di-*C*-*β*-D-glucoside	Flavonoids

10	46.316	C_25_H_24_O_12_	515.1201^a^	515.1195	1.2	191.0566, 179.0356, 335.0887, 515.1225, 135.0461, 173.0463, 161.0243	1, 3-DiCQA^d^	Organic acids
			551.0970^b^	551.0962	1.5	353.0893, 515.1234, 191.0568, 179.0357, 335.0785, 173.0443, 135.0461		

11	48.753	C_27_H_30_O_16_	609.1456^a^	609.1461	-0.8	609.1480, 463.0192, 447.0915, 301.0339	Isomer of rutin	Flavonoids
			645.1207^b^	645.1228	-3.2	609.1482, 463.0906, 447.0931, 301.0316		

12	55.161	C_27_H_30_O_16_	609.1475^a^	609.1461	2.3	609.1516, 301.0362	Rutin^d^	Flavonoids

13	55.657	C_21_H_20_O_12_	463.0890^a^	463.0882	1.7	463.0914, 301.0359	Hyperoside^d^	Flavonoids

14	56.590	C_21_H_20_O_12_	463.0896^a^	463.0882	3.0	463.0937, 301.0373, 179.0010	Isoquercetin^d^	Flavonoids

15	57.238	C_21_H_20_O_11_	447.0945^a^	447.0933	2.7	285.0404, 447.0940	Kaempferol-3-*O*-glucoside	Flavonoids

16	57.638	C_27_H_30_O_15_	593.1510^a^	593.1512	-0.3	593.1525, 285.0469	Kaempferol-3-*O*-rutinoside	Flavonoids

17	61.037	C_21_H_20_O_11_	447.0939^a^	447.0933	1.4	447.0974, 285.0420	Quercetin-3-*O*-rhamnoside	Flavonoids
			483.0705^b^	483.0700	1.1	447.0954, 285.0406		

18	61.495	C_25_H_24_O_12_	515.1220^a^	515.1195	4.9	191.0579, 353.0917, 179.0364 515.1287	3, 4-DiCQA^d^	Organic acids

19	61.913	C_25_H_24_O_12_	515.1212^a^	515.1195	3.3	191.0572, 353.0909, 179.0353, 173.0456, 135.0454, 515.1298	3, 5-DiCQA^d^	Organic acids

20	64.996	C_25_H_24_O_12_	515.1215^a^	515.1195	3.9	173.0462, 353.0913, 179.0356, 515.1294, 191.0572, 135.0448	4, 5-DiCQA^d^	Organic acids

Quasimolecular ion was [M-H]^−^. ^b^Quasimolecular ion was [M* *+* *Cl]^−^. ^c^Sequencing according to the abundance. ^d^Confirmed by comparison with reference substances.

**Table 5:** The contents of the 13 components in 31 batches of *A. capillaris* (mg/g).

No.	1-CQA	NCA	CA	Caffeic acid	CCA	*p*-Hydroxyacetophenone	1, 3-DiCQA	Rutin	Hyperoside	Isoquercetin	3, 4-DiCQA	3, 5-DiCQA	4, 5-DiCQA	Total
S1	1.357	0.197	8.575	0.236	0.358	0.389	0.130	0.150	0.556	0.299	0.279	5.857	1.564	19.946
S2	1.175	0.248	7.646	0.216	0.430	0.337	0.153	0.099	0.390	0.274	0.451	5.038	1.743	18.199
S3	1.076	0.247	7.601	0.242	0.396	0.367	0.124	0.132	0.506	0.364	0.390	6.015	2.023	19.482
S4	1.007	0.259	5.369	0.194	0.417	0.709	0.398	0.086	0.200	0.101	0.348	4.575	1.986	15.649
S5	0.632	0.144	3.124	0.202	0.259	0.174	0.180	0.071	0.126	0.103	0.241	2.834	1.153	9.242
S6	0.600	0.150	3.177	0.199	0.266	0.186	0.169	0.065	0.124	0.100	0.173	2.655	1.170	9.036
S7	0.861	0.153	4.258	0.207	0.269	0.301	0.074	0.092	0.279	0.225	0.181	2.778	1.155	10.832
S8	0.680	0.161	3.463	0.214	0.287	0.165	0.191	0.078	0.133	0.103	0.258	3.139	1.353	10.225
S9	0.446	0.154	3.535	0.232	0.255	0.147	0.056	0.062	0.242	0.170	0.203	2.880	1.132	9.515
S10	0.990	0.223	7.134	0.251	0.373	0.330	0.059	0.085	0.453	0.334	0.298	4.335	1.522	16.388
S11	1.271	0.268	6.936	0.190	0.444	0.827	0.332	0.106	0.259	0.108	0.417	5.561	1.929	18.647
S12	1.043	0.246	5.317	0.189	0.435	0.511	0.117	0.078	0.282	0.150	0.343	4.370	1.910	14.992
S13	0.658	0.140	5.505	0.086	0.284	0.236	0.059	0.136	0.276	0.122	0.254	3.952	0.948	12.656
S14	0.897	0.229	7.479	0.103	0.510	0.317	0.103	0.108	0.351	0.114	0.289	5.095	1.645	17.241
S15	0.647	0.129	3.402	0.113	0.225	0.365	0.105	0.085	0.130	0.075	0.186	2.407	0.952	8.820
S16	0.555	0.101	3.704	0.095	0.217	0.246	0.056	0.097	0.158	0.085	0.109	2.462	0.674	8.556
S17	1.189	0.150	7.572	0.112	0.262	0.379	0.057	0.122	0.561	0.251	0.257	4.794	1.013	16.718
S18	0.630	0.145	4.387	0.086	0.271	0.259	0.106	0.126	0.178	0.081	0.243	3.825	1.202	11.536
S19	1.072	0.150	4.875	0.208	0.282	0.151	0.069	0.063	0.150	0.099	0.269	4.574	1.178	13.139
S20	0.962	0.205	6.458	0.099	0.380	0.510	0.132	0.090	0.267	0.110	0.343	5.388	1.548	16.493
S21	1.065	0.245	6.018	0.188	0.426	0.338	0.093	0.116	0.361	0.179	0.527	6.191	2.155	17.902
S22	0.929	0.283	7.567	0.085	0.502	0.265	0.078	0.108	0.217	0.106	0.558	7.919	2.066	20.683
S23	0.550	0.171	4.541	0.096	0.265	0.296	0.060	0.106	0.273	0.142	0.129	2.844	0.803	10.276
S24	1.028	0.195	6.168	0.132	0.345	0.521	0.170	0.120	0.311	0.146	0.340	5.419	1.521	16.416
S25	1.272	0.164	6.073	0.139	0.278	0.125	0.333	0.079	0.205	0.151	0.459	4.780	1.299	15.356
S26	0.810	0.177	5.326	0.123	0.356	0.307	0.098	0.133	0.246	0.100	0.269	4.962	1.426	14.334
S27	0.783	0.117	3.359	0.157	0.210	0.104	0.173	0.090	0.145	0.090	0.246	3.141	0.952	9.568
S28	0.879	0.296	6.849	0.206	0.476	0.437	0.104	0.167	0.532	0.211	0.502	6.732	2.456	19.847
S29	0.796	0.203	5.721	0.135	0.261	0.273	0.099	0.132	0.509	0.227	0.297	4.595	1.162	14.411
S30	0.862	0.159	4.057	0.141	0.239	0.352	0.245	0.066	0.176	0.093	0.246	3.280	1.208	11.123
S31	1.379	0.171	11.377	0.079	0.381	0.341	0.017	0.060	0.288	0.144	0.285	6.786	0.815	22.124
Average	0.907	0.190	5.696	0.160	0.334	0.331	0.134	0.100	0.287	0.157	0.303	4.490	1.408	14.495

**Table 6:** Bivariate correlation analysis results of the correlation between the contents of CA and 12 other components and the total of all 13 components in 31 batches of *A. capillaris*.

Component	Correlation coefficient of Spearson	*P*
1-CQA	0.748	0.0001
NCA	0.665	0.0001
Caffeic acid	−0.340	0.856
CCA	0.698	0.0001
*p*-Hydroxyacetophenone	0.529	0.002
1,3-DiCQA	−0.160	0.390
Rutin	0.444	0.012
Hyperoside	0.797	0.0001
Isoquercetin	0.650	0.0001
3,4-diCQA	0.696	0.0001
3,5-diCQA	0.861	0.0001
4,5-diCQA	0.498	0.005
Total of 13	0.942	0.0001

## Data Availability

The data used to support the findings of this study are included within the article.
